# Hospitals admitting at least 100 patients with stroke a year should have a stroke unit: a case study from Australia

**DOI:** 10.1186/s12913-017-2150-2

**Published:** 2017-03-16

**Authors:** Dominique A. Cadilhac, Monique F. Kilkenny, Nadine E. Andrew, Elizabeth Ritchie, Kelvin Hill, Erin Lalor, On behalf of the Stroke Foundation National Advisory Committee: and the National Stroke Audit Collaborative

**Affiliations:** 1 Stroke and Ageing Research, School of Clinical Sciences at Monash Health, Department of Medicine, Monash University, Clayton, 3168 Vic Australia; 2 The Florey Institute of Neuroscience and Mental Health, Stroke Division, Heidelberg, 3081 Vic Australia; 3 Stroke Foundation, Melbourne, 3000 Vic Australia

**Keywords:** Audit, Stroke, Stroke unit, Processes of care, Thrombolysis

## Abstract

**Background:**

Establishing a stroke unit (SU) in every hospital may be infeasible because of limited resources. In Australia, it is recommended that hospitals that admit ≥100 strokes per year should have a SU. We aimed to describe differences in processes of care and outcomes among hospitals with and without SUs admitting at least 100 patients/year.

**Methods:**

National stroke audit data of 40 consecutive patients per hospital admitted between 1/7/2010-31/12/2010 and organizational survey for annual admissions were used. Descriptive analyses and multilevel regression were used to compare patient outcomes. Sensitivity analysis including only hospitals meeting all of the Australian SU criteria (e.g., co-location of beds; inter-professional team; weekly meetings; regular training) was performed.

**Results:**

Two thousand eight hundred ninety-eight patients from 72/108 eligible hospitals completing the audit (SU = 60; patients: 2,481 [mean age 76 years; 55% male] and non-SU patients: 417 [mean age 77; 53% male]). Hospitals with SUs had greater adherence to recommended care processes than non-SU hospitals. Patients treated in a SU hospital had fewer new strokes while in hospital (OR: 0.20; 95% CI 0.06, 0.61) and there was a borderline reduction in the odds of dying in hospital compared to patients in non-SU hospitals (OR 0.57 95%CI 0.33, 1.00). Among SU hospitals meeting all SU criteria (*n* = 59; 91%) the adjusted odds of having a poor outcome was further reduced compared with patients attending non-SU hospitals.

**Conclusion:**

Hospitals annually admitting ≥100 patients with acute stroke should be prioritized for establishment of a SU that meet all recommended criteria to ensure better outcomes.

## Background

Stroke is a worldwide health-care problem in many countries because it is a leading cause of death and major cause of adult disability [1, 2]. Stroke unit care has been long recognized as the major component of providing effective stroke services for reducing death and disability after stroke. Stroke units have been shown to benefit all type of patients irrespective of age, severity or stroke type (ischemic or hemorrhagic) [3, 4]. There is also evidence that stroke unit care is cost-effective [5].

The World Stroke Organization has recently released guidelines and a quality action plan framework to inform stroke policy and set strategic directions to improve the standards of stroke care around the world [6]. In Australia, the Stroke Foundation developed an Acute Stroke Services Framework as part of a strategy to improve the quality of acute stroke care services in Australia [7]. In brief, the main features and minimum criteria for an acute stroke unit as described in this framework are co-location of beds; having an inter-professional team that meets weekly to discuss patient care; and regular staff education and training (Table 1) [7]. The framework can be used to identify where acute stroke units should be located based on the number of patients admitted per year. That is, within Australia all hospitals admitting more than 100 patients with acute stroke per year (equivalent to 2 to 3 admissions per week) should have a stroke unit. There is some evidence to support this criterion whereby patients managed in hospitals treating <50 strokes per year (considered a small number of admissions) was associated with greater stroke mortality than in those treated in hospitals with more admissions [8–11]. Further evidence is required to determine whether the difference in mortality or other adverse outcomes between hospitals with similar large admission numbers of patients is due to differences in patient characteristics or because of the more specialized and coordinated care that is provided in a stroke unit.

**Table 1:** Stroke unit definition from Acute Stroke Services Framework

	All hospitals		Large hospitals (100+ patients/year)	
Does your hospital have a specialized stroke unit?^a^	Yes *N* = 74 n (%)	No *N* = 114 n (%)	Yes *N* = 65 n (%)	No *N* = 16 n (%)
Minimum criteria				
1. Co-located beds within a geographically defined unit.	72 (97)	13 (11)	64 (98)	3 (19)
2. Dedicated, interprofessional team with members who have a special interest in stroke and/or rehabilitation. The minimum team would consist of medical, nursing and allied health (including OT, PT, SP, SW & DT)	69 (93)	27 (24)	61 (94)	9 (56)
3. Interprofessional team meets at least once per week to discuss patient care.	72 (97)	69 (61)	64 (99)	15 (94)
4. Regular programs of staff education and training relating to stroke, (e.g., dedicated stroke inservice program and/or access to annual national or regional stroke conference)	70 (95)	35 (31)	61 (94)	7 (44)
Hospitals meets all minimum criteria listed above (1–4)	66 (89)	3 (3)	59 (91)	2 (13)

*OT* Occupational therapist, *PT* physiotherapist, *SP* Speech pathologist, *SW* Social worker, *DT* Dietician

^a^self-reported from the acute services organizational survey [19] Source: adapted from Stroke Foundation, Acute Service Framework [9]

The aims of this study were to (a) describe current access to acute stroke units in Australia by number of annual admissions; and (b) determine the differences in adherence to processes of care and in-hospital outcomes among hospitals with and without stroke units admitting at least 100 patients per year. Our primary hypothesis was that patients admitted to hospitals with at least 100 stroke admissions without a stroke unit would not provide the same quality of care when compared to those with a stroke unit. As a sensitivity analysis, we also compared these hospitals with and without stroke units (self-reported) that admit at least 100 patients per year based on whether they met all the Acute Stroke Services Framework criteria. This latter analysis would support the utility for having established criteria for hospitals.

## Methods

We used an observational study design utilizing cross-sectional, consecutive medical record data obtained from the 2011 Stroke Foundation Acute Services Audit Program. The aims of this biennial audit are to monitor the quality of care and outcomes of patients with stroke admitted to mainly public hospitals [12, 13]. There are two components of this program: an organizational survey completed by a clinician with the best knowledge of the stroke service and hospital; and a clinical audit of patient medical records. All Australian hospitals that admit and manage patients with acute stroke were approached to participate. Clinical audit data were collected retrospectively for the first 40 or more consecutive acute stroke admissions presenting to hospital between 01/07/2010 and 31/12/2010. The audit was performed by trained data abstractors using a validated web-based data entry tool. Data collected include: demographic characteristics; history of risk factors; stroke severity measures as per the validated Counsell and colleagues simple variables model (such as ability to walk on admission, incontinence within 72 h, arm deficit on admission and Speech/communication deficit on admission) [14] process of care indicators to measure adherence to the Australian Stroke Clinical guidelines [15] (such as stroke unit care, aspirin within 48 h and assessments by allied health) and health outcomes (such as discharge destination, dependence at discharge and death). Recording of outcomes, such as a new stroke or stroke progression while in hospital for the index admission, was based on the documentation in the medical record.

Patients with an ICD10 code of I61.0 - I61.9 (intracerebral hemorrhage), I63.0 - I63.9 (cerebral infarction), I64 (stroke not specified as hemorrhage or infarction) and I62.9 (nontraumatic intracranial hemorrhage, unspecified) were eligible for inclusion in the clinical audit.

### Data access

Chief executive officers and the Heads of the Stroke Service from eligible hospitals were invited to formally participate in the audit program by the Stroke Foundation. Participation was voluntary with national and state-level aggregated data presented in a national report and a site report specifically provided for the local use of hospital staff to review and improve the quality of routine stroke care. No data are publically presented which could identify an individual hospital. Following confirmation of agreement to participate, data were collected by hospital staff and submitted to the Stroke Foundation via the secure web-based data collection tool. Data collected did not include patient identifiers. No written informed consent for participation in the study was obtained from patients with stroke to avoid selection bias since the data were retrospectively obtained and were anonymized [16].

### Definition of stroke unit status

In this study hospitals were classified as having a stroke unit if they reported in the organizational survey that their hospital had a specialized stroke unit. In addition, we mapped the adherence to the minimum criteria for stroke units as described in the Acute Stroke Services Framework [7] by self-reported stroke unit status (Table 1).

### Eligibility criteria for hospitals to be included in the analysis

Large hospitals that participated in the 2011 clinical audit were selected as the unit of analysis. In this paper, classification for being a large hospital was based on self-report of the number of annual admissions being 100 or more patients from the Stroke Foundation organizational survey. This excluded seven hospitals from the clinical audit in 2011 with annual admissions less than 100 (median admissions = 61) that had a stroke unit (total patients excluded 650; admitted on the stroke unit *n* = 93 < 3%) (Table 2)*.*

**Table 2:** Resources and protocols to support evidence-based care and patient processes of care by number of annual stroke admissions

Annual stroke admissions	<50	50–99	100–199	≥200
Organizational survey (*N* = 188)	n (%)	n (%)	n (%)	n (%)
Number of hospitals participated	*N* = 81	*N* = 26	*N* = 34	*N* = 47
Median (Q1, Q3) patient admissions with stroke	12 (5,25)	67 (53,80)	142 (115,164)	326 (250,465)
Number of self-reported stroke units	2 (3)	7 (27)	20 (59)	45 (96)
Ambulance arrangements	32 (40)	7 (27)	11 (32)	28 (60)
ED protocols for rapid triage	41 (51)	16 (62)	23 (68)	41 (87)
Access on site MRI	25 (31)	14 (54)	30 (88)	47 (100)
Offering thrombolysis	8 (10)	5 (19)	14 (41)	41 (87)
Assessments rehabilitation	36 (44)	18 (69)	24 (71)	39 (83)
Education – staff stroke	22 (27)	15 (58)	24 (71)	44 (94)
Clinical audit (*N* = 108)^a^				
Number of patient records audited	*N* = 196	*N* = 454	*N* = 1,040	*N* = 1,858
Number of stroke units (self-reported in organizational survey)	2	5	18	42
Treated in a stroke unit: n (%)	15 (8)	78 (17)	504 (49)	1,477 (80)

*Q1* 25^th^ percentile, *Q3* 75^th^ percentile, *ED* Emergency Department, *MRI* magnetic resonance imaging

^a^these hospitals contributed clinical audit data in addition to organizational survey responses

### Data analyses

Univariable analyses were used to compare the patient characteristics and process of care indicators and outcomes of patients with stroke admitted to a stroke unit hospital and non-stroke unit hospital. Chi-square tests were used for categorical variables and the Wilcoxon Mann-Whitney Rank Sum test for continuous variables.

To assess differences in patient discharge outcomes we used multivariable analyses. Random effects, multi-level logistic regression modeling with one level defined as the hospital unit, to account for correlations between patients that were managed within an individual hospital; and the other as the patients as individual units were used. The dependent variable was the health outcome (e.g., dependent at discharge, new stroke, stroke progression, discharge destinations, died in hospital or died in hospital within 7 days) and the independent variables were hospital stroke unit status, age, sex, stroke severity variables (using the simple variables model [14]), independent prior to stroke and type of stroke (e.g., ischemic, intracerebral hemorrhage or unknown subtype). We used two regression models to investigate differences in outcomes for patients based on whether or not their hospital: a) self-reported having a stroke unit; and b) met all the Acute Stroke Services Framework stroke unit criteria. The latter model was a sensitivity analysis to compare outcomes of patients admitted to a stroke unit hospital which met the stroke unit criteria and non-stroke unit hospitals that did not meet the criteria.

Unadjusted and adjusted odds ratio and 95% confidence intervals (95% CIs) were calculated. Independent variables were selected based on known confounders and statistical significance from the initial univariable analyses. Logistic regression analysis was used to calculate differences in the risk of mortality at seven days to provide information at a common time point since end-of-life care and transfer practices for palliation may vary between hospitals, therefore examining only in-hospital deaths which may not occur at a fixed point in time could be misleading. Because there was an imbalance in the frequency of documentation of unknown stroke subtype between stroke unit and non-stroke unit hospitals, in a further sensitivity analysis we assumed that unknown stroke type was equivalent to being an ischemic event (since over 90% of patients had brain imaging and it may have been an administrative discharge coding issue) and combined these two groups and repeated the analysis of health outcomes related to care in hospital to see whether the results changed. We also explored the influence of access to the stroke unit on the same outcomes among the large stroke unit hospitals, since not all patients will be admitted into the stroke unit. Stata (Version 10.1, StataCorp, College Station, TX, 2010) statistical software was used for all analyses and a *p*-value of < 0.05 was considered a significant difference.

## Results

There were 188 hospitals that responded to the organizational survey (*n* = 25,597 annual stroke admissions): 74 (39%) reported having a specialized stroke unit. The majority of hospitals with a stroke unit met the minimum criteria (Table 1). There were 81 hospitals classified as large hospitals that admitted at least 100 patients per year (*n* = 22,555 annual stroke admissions [88% of total cohort among which 16 did not have a stroke unit. The proportion of hospitals with a stroke unit increased as the annual admissions of hospitals increased (Table 2). There were significant differences between the use of resources and protocols to support evidence based care between hospitals with different numbers of annual stroke admissions (Table 2).

Among the 3,548 patients cases contributed from 108 hospitals in the clinical audit there were 2,898 patients (82%) admitted to a large hospital. The majority of these patients were from hospitals with a stroke unit (*n* = 2,481 from 60 hospitals ≥ 100 stroke admissions per year) compared with non-stroke unit hospitals (*n* = 417 from 12 hospitals). There were differences in patient factors, such as being more independent prior to admission and having less severity at time of admission in terms of communication problems and incontinence, for those admitted to a stroke unit hospital compared with patients admitted to a non-stroke unit hospital (Table 3).

**Table 3:** Patient characteristics by self-reported stroke unit status with at least 100 admissions in a year

Patient characteristics	SU hospital n (%) *N* = 2,481	No SU hospital n (%) *N* = 417	*p*-value*
Age (years), median (Q1,Q3)	76 (65,84)	77 (66,85)	0.07
Male	1,364 (55)	222 (53)	0.5
Lived at home prior to stroke	2,233 (90)	358 (86)	0.01
Type of stroke			
Ischemic stroke	1,983 (82)	271 (69)	<0.001
Intracerebral hemorrhage	358 (15)	61 (15)	0.7
Unknown	69 (3)	62 (16)	<0.001
Previous stroke/transient ischemic attack	792 (32)	157 (38)	0.02
Modified Rankin Score (0–1)^a^	1,441 (62)	164 (52)	0.001
IHD/recent AMI	650 (33)	117 (37)	0.1
Diabetes	590 (29)	99 (29)	1.0
Atrial fibrillation	683 (34)	114 (38)	0.2
Stroke severity variables on admission (adapted from Counsell et al. [14])			
Arm deficit	1,655 (69)	277 (71)	0.5
Unable to walk	1,575 (64)	242 (62)	0.3
Incontinent within 72 h	900 (38)	160 (45)	0.007
Speech/communication deficit	1,461 (62)	265 (70)	0.003

*Q1* 25th percentile, *Q3* 75th percentile, *SU* stroke unit, *IHD* ischemic heart disease, *AMI* acute myocardial infarction

*Chi square test *p* value for difference between hospitals with a stroke unit and those without a stroke unit

^a^This score is used to identify patients that may be considered ‘independent’ prior to stroke onset where they have either no symptoms or no significant disability despite symptoms; able to carry out all usual duties and activities [29]

**Fig. 1:**
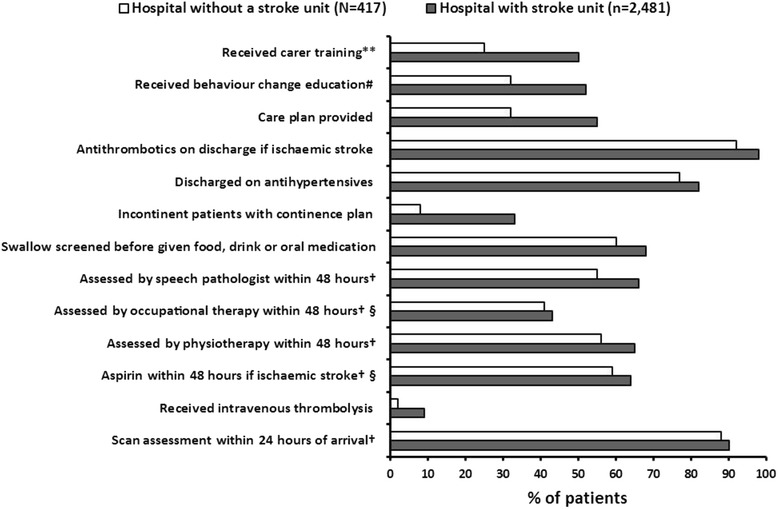
Adherence to processes of care in hospitals admitting at least 100 patients with stroke per year by self-reported stroke unit status. §*p* value >0.05 (*not significant*) for difference between hospitals with a stroke unit and those without a stroke unit; †Where times were available; #Education about behavior change for modifiable risk factors prior to discharge; **Includes only those who were eligible for training or assessment

Non-stroke unit hospitals had reduced adherence to important processes of care known to improve patient outcomes when compared to those with a stroke unit (Fig. 1). This included providing intravenous thrombolysis, assessment by allied health, swallow screen before food or drink and discharge care plans. In stroke unit hospitals over 80% of patients received stroke unit care at some stage of their admission for acute stroke.

Based on univariable analysis, patients with stroke admitted to a stroke unit hospital had reduced odds of dying, or having another stroke while in hospital (Table 4). The median ‘days to death’ in hospital was 4 days (25^th^ percentile 2 days and 75^th^ percentile 9 days).

**Table 4:** Outcomes of care in hospitals admitting at least 100 patients in a year by stroke unit status

Outcomes Reference: stroke unit hospital versus no stroke unit hospital	SU hospital n (%) *N* = 2,481	Non-SU hospital n (%) *N* = 417	Self-reported SU status *N* = 81 Odds ratio (95% CI)^a^	Meet all SU criteria *N* = 59 Odds ratio (95% CI)^a^
Modified Rankin Score (mRS) on discharge 0–2 (i.e., none to slight disability) [29]	885 (36)	106 (25)*	1.09 (0.65, 1.84)	0.94 (0.59, 1.52)
Stroke progression (including hemorrhagic transformation)	256 (10)	34 (8)	1.28 (0.67, 2.47)	1.14 (0.64, 2.04)
New stroke (recurrent event in hospital)	42 (2)	35 (8)*	0.20 (0.06, 0.61)*	0.25 (0.08, 0.74)*
Discharge destination^b^				
Discharged home	985 (45)	155 (45)	0.84 (0.48, 1.50)	0.88 (0.52, 1.50)
Discharged to inpatient rehabilitation	859 (35)*	98 (24)	1.23 (0.77, 1.96)	1.18 (0.77, 1.80)
Discharged to an aged care facility	219 (10)	51 (15)*	0.72 (0.36, 1.46)	0.70 (0.37, 1.34)
Died in hospital	291 (12)	75 (18)*	0.57 (0.33, 1.00)**	0.51 (0.31, 0.83)*
Died or discharged to aged care facility	510 (21)	126 (30)*	0.61 (0.36, 1.02)	0.58 (0.36, 0.92)*

^a^Each outcome was adjusted for hospital stroke unit status (self-reported or if all the Acute Stroke Services Framework stroke unit criteria were met^9^), age, sex, stroke severity variables (e.g., unable to walk on admission), independent prior to stroke and type of stroke (ischemic, intracerebral hemorrhage or unknown type)

^b^Excludes discharge destination noted as a statistical discharge (11% of patients); *p < 0.05; **p < 0.07

Patients admitted to a stroke unit hospital were also less likely to be dependent at discharge or to be discharged to an aged care facility, than patients admitted to a non-stroke unit hospital. There was no difference in being discharged home between patients admitted to a stroke unit or non-stroke unit hospital. In addition, there was no difference in patients experiencing complications such as fever and urinary tract infection for patients admitted to a stroke unit or non-stroke unit hospital.

Multivariable analyses showed that patients admitted to stroke unit hospitals (based on self-reported stroke unit status) were less likely to have a new stroke (another stroke event while in hospital) and had reduced odds of dying in hospital compared to patients in non-stroke unit hospitals. Patients who were treated in a hospital with a stroke unit had a borderline non-significant reduced risk of death up to seven days after admission (OR: 0.57; 95% CI 0. 28, 1.15) when compared to patients who were treated in a hospital without a stroke unit. There was no association with level of disability at time of discharge by self-reported stroke unit hospital status (Table 4).

In our sensitivity analysis where we reclassified unknown stroke type as ischemic, the results remained similar for outcomes of new stroke (OR 0.17; 95% CI 0.06, 0.52) or died while in hospital (OR 0.60; 95% CI 0.34, 1.04). For the sensitivity analysis where outcomes for hospitals reporting having a stroke unit that met all the minimum criteria of the Acute Stroke Service Framework compared to non-stroke unit hospitals that did not meet the criteria, we found stronger signals for better outcomes being achieved including a reduced odds of death or being discharge to aged care (OR 0.58; 95% CI 0.36, 0.92). In contrast, a slightly higher odds for fewer new strokes during hospitalization (OR 0.25; 95% CI 0.08, 0.74) was noted using the criteria based definition compared with self-reported stroke unit status, but this results still indicated fewer cases of in-hospital stroke were likely (Table 4).

Lastly, when we explored the influence of access to the stroke unit on in-hospital outcomes among the large stroke unit hospitals the likelihood of better outcomes were stronger including having a greater odds of being independent at time of discharge or being discharged to a rehabilitation facility (Table 5).

**Table 5:** Outcomes of care in stroke unit hospitals admitting at least 100 patients in a year by stroke unit access status

Outcomes Reference: stroke unit admission versus stroke unit non-admission *N* = 2043 patients	Admission into a SU^a^ *N* = 60 hospitals Odds ratio (95% CI)^b^	Met all SU criteria *N* = 59 hospitals Odds ratio (95% CI)
Modified Rankin Score (mRS) on discharge 0–2 (i.e., none to slight disability) [29]	1.46 (0.98, 2.18)**	1.63 (1.08, 2.46)*
Stroke progression (including hemorrhagic transformation)	0.63 (0.41, 0.96)*	0.63 (0.41, 0.98)*
New stroke (recurrent event in hospital)	0.36 (0.18, 0.74)*	0.38 (0.18, 0.81)*
Discharge destination^c^		
Discharged home	1.17 (0.79, 1.75)	1.32 (0.88, 2.00)
Discharged to inpatient rehabilitation	2.29 (1.65, 3.18)*	2.39 (1.71, 3.37)*
Discharged to an aged care facility	0.59 (0.35, 1.01)	0.59 (0.35, 1.01)
Died in hospital	0.20 (0.13, 0.31)*	0.22 (0.14, 0.35)*
Died or discharged to aged care facility	0.28 (0.19, 0.41)*	0.29 (0.19, 0.44)*

^a^self-reported from the organizational survey

^b^Each outcome was adjusted for stroke unit admission status, age, sex, stroke severity variables (e.g., unable to walk on admission), independent prior to stroke and type of stroke (ischemic, intracerebral hemorrhage or unknown subtype)

^c^Excludes discharge destination noted as a statistical discharge (11% of patients); *p < 0.05; **p < 0.07

## Discussion

Stroke unit care should be available to all patients with stroke since it reduces death and disability, and is cost effective [4, 17]. For this reason, stroke unit access remains one of the most important recommendations in clinical guidelines [15, 18]. However, in some countries around the world, it may be infeasible to have a stroke unit in every hospital [6]. Methods to prioritize where acute stroke units should be established based on number of stroke admissions and meeting minimum criteria as illustrated here from the Australian Acute Stroke Services Framework are important [7].

In developing countries the use of organized stroke units is tending to increase over time. In Australia, between 1999 and 2013 the number of acute stroke units has increased from 35 to 92 serving a geographically dispersed population of approximately 24 million people [19–21]. By country, the reported proportion of patients treated in stroke units range from 25% in Thailand, 58% in Australia and Canada, 88% in the UK, to greater than 85% in the Sweden [22–25]. Therefore, access remains an issue. As reported here, even within hospitals that have a stroke unit, the bed numbers may be insufficient to meet demand since one in five patients was not managed in the stroke unit. In a secondary analysis, we found that patients that were admitted into a stroke unit in the large stroke unit (based on self-reported status and SU criteria) hospitals had better outcomes consistent with what is reported in the literature.

As described in Table 2, in Australia the number of hospitals with a stroke unit increased as the annual admissions of patients with stroke increased. Hospitals treating at least 100 patients with stroke per year tended to have more organized systems and infrastructure (i.e., specialist staff such as neurologists and access to imaging on-site) that provide guidance for whether a stroke unit should exist at a hospital.

Multivariable logistic regression analyses were performed in an attempt to assess potential differences in outcomes for patients treated in hospitals admitting at least 100 patients with stroke per year that did and did not have a stroke unit. The statistical models included variables commonly known to influence the likelihood of a poor outcome, and included accounting for potential correlations within individual hospitals. We found better patient outcomes, such as reducing new strokes and deaths in hospital, for hospitals with a stroke unit compared to those without that admit at least 100 patients per year.

Comparison of hospitals reporting to have a stroke unit and whether they met the minimum criteria for a stroke unit highlighted differences in self-report and understanding of what constitutes a stroke unit based on the Acute Stroke Service Framework. We found that seven hospitals reported having a stroke unit without meeting all the criteria and three hospitals met the criteria without claiming to have a stroke unit. Sensitivity analysis of large hospitals with a stroke unit that met the criteria and non-stroke unit hospitals that did not meet the criteria provided stronger evidence of the possibility of achieving better patient outcomes in terms of fewer new strokes, deaths in hospital or being discharge to an aged care facility. Therefore, all hospitals with stroke units should aim to conform to these criteria.

Within the area of stroke very few studies have investigated the issue of hospital patient admission volumes and outcome [8–11]. Although not directly comparable since we included all major stroke types and could distinguish between hospitals with and without stroke units and the number of patients getting access to these units, our work is complementary to that of Hall and colleagues from Canada. These authors found that having between 100 and 165 ischemic stroke admissions were the break points associated with lower mortality rates within 30 days [8]. Importantly, these authors emphasized the need for more specialized centers when there are larger patient volumes to ensure access to stroke units and sustainable interprofessional teams [8]. In this paper we clearly illustrate the importance of patients getting access to the stroke unit when attending a large hospital with a stroke unit.

While the reasons behind the benefits of stroke unit care are not completely understood it is clear that relevant expertise and experience is fundamental [26]. Larger numbers of patients with stroke enables staff to gain this expertise and experience, which in hospitals with fewer admissions may not be possible. In our study patients with stroke admitted to a large hospital, that treated at least 100 patients per year without a stroke unit were less likely to receive important clinical processes of care such as thrombolysis, aspirin within 48 h if ischemic stroke patient, treatment from allied health staff or be discharged on appropriate prevention medications compared with patients admitted to a hospital with a stroke unit.

Time is critical for stroke and there are interventions such as thrombolysis that are effective if given in the early stages of stroke [27, 28]. Arrangements with ambulance services and protocols in emergency departments for rapid triage for stroke are essential. The use of protocols for rapid assessment and management is important in hospitals admitting large numbers of patients with stroke [15]. Our data provides evidence that many of the non-stroke unit hospitals were less likely to have these organizational features compared with stroke unit hospitals. The reported use of protocols to guide assessment of common impairments after stroke was more common in hospitals with stroke units than in those without stroke units. Targeted programs are required in order to implement these organizational processes and protocols in these large hospitals without a stroke unit in order to improve early treatment after the onset of stroke.

More recently the Australian Stroke Coalition have proposed a change to the Acute Stroke Services Framework [7] in relation to the criteria for establishment of stroke units. In the current framework, the recommended number of admissions for establishment of a stroke unit is at least 100 patients per year. Subsequent to this recommendation, the proportion of patients presenting to such hospitals with a stroke unit has increased from 86% (2011 audit) [24] to 94% in the 2013 audit [21]. There is a proposal now to reduce the minimum number of admissions to a least 75 patients per year [29]. Further research to understand the implication of this change is needed using similar approaches to what has been outlined in this paper.

In this paper we have provided quantitative justification for why it is important that stroke units are established in hospitals admitting at least 100 patients with stroke per year. In addition, these hospitals are required to provide appropriate processes and adequate resources, including access to stroke unit beds, if the benefits to patients are to be realized. A cornerstone for improving stroke care in Australia is increasing the number of stroke units in hospitals admitting large numbers of patients in all geographical locations. We acknowledge that where specialist resources are limited it can be difficult to establish a stroke unit [6]. We have been able to show, in complementary research, that investment in Stroke Clinical Coordinators who implemented organizational change, together with increased clinician resources, effectively improved stroke care in rural hospitals resulting in more patients being discharged home [30]. These findings are relevant to countries which do not have universal access to stroke units and show that investment in leadership roles is worthwhile.

The strengths of this study include the use of comprehensive national data that has provided a large sample of patients with and without access to a stroke unit, from a wide cross-section of Australian hospitals [24]. There are also some limitations that must be acknowledged. Adherence to recommended management may have been under-reported since data were retrospectively abstracted from medical records and subject to reporting biases. Steps to minimize bias in this audit included verifying in a subsample inter-rater reliability, providing detailed and consistent auditor training and supplying a comprehensive data dictionary. Sampling bias was minimized by providing a defined period for inclusion of cases and requesting consecutive admissions. Nonetheless, the number of ischemic strokes in the stroke unit group (82%) was greater than in the non-stroke unit group (69%) while the number of unknown stroke types was larger in the non-stroke unit group (16%) than in the stroke unit group (3%). The differences in recording of stroke type may have affected the results of our multivariable analyses. However, our main findings for patient outcome would have been similar based on the results obtained from the sensitivity analysis presented for this issue. Lastly, there was no date and time information collected on when the patients experienced their outcomes while in hospital and there was no formal adjudication process in the event of unclear events.

## Conclusions

Hospitals admitting at least 100 strokes per year treat the majority of patients with stroke in Australia. The hospitals without a stroke unit should be prioritized for establishment of a stroke unit to improve that quality of care that is known to lead to better patient outcomes. Ensuring stroke units meet the expected criteria for an organized stroke service and have the capacity to treat the patients with stroke was shown in this study as being essential to achieving greater patient outcomes.

## Abbreviations

95% CI: 95% confidence interval
ED: Emergency department
MRI: Magnetic resonance imaging
SU: Stroke unit
